# Dependence of image quality of LGE-MRI of the left atrium on the acceptance rate of respiratory navigator and acquisition time

**DOI:** 10.1186/1532-429X-15-S1-P269

**Published:** 2013-01-30

**Authors:** Sathya Vijayakumar, Chankevin Tek, Nathan S Burgon, Christopher McGann, Nassir F Marrouche, Eugene Kholmovski

**Affiliations:** 1 CARMA Center, Dept. of Cardiology, University of Utah, Salt Lake City, UT, USA; 2 UCAIR, Dept. of Radiology, University of Utah, Salt Lake City, UT, USA

## Background

Atrial fibrillation (AF) is a common sustained cardiac arrhythmia associated with increased risks of heart failure and stroke. Late gadolinium enhanced MRI (LGE-MRI) is being routinely used to assess and manage the treatment of AF. To acquire LGE images of thin left atrial (LA) wall high-resolution 3D inversion recovery prepared GRE pulse sequence with cardiac gating and respiratory navigation should be used. This makes the image quality dependent on the patient's heart rate and respiration pattern. In this work, we study the effect of the patient respiration on image quality of LGE-MRI of LA.

## Methods

**Figure 1:**
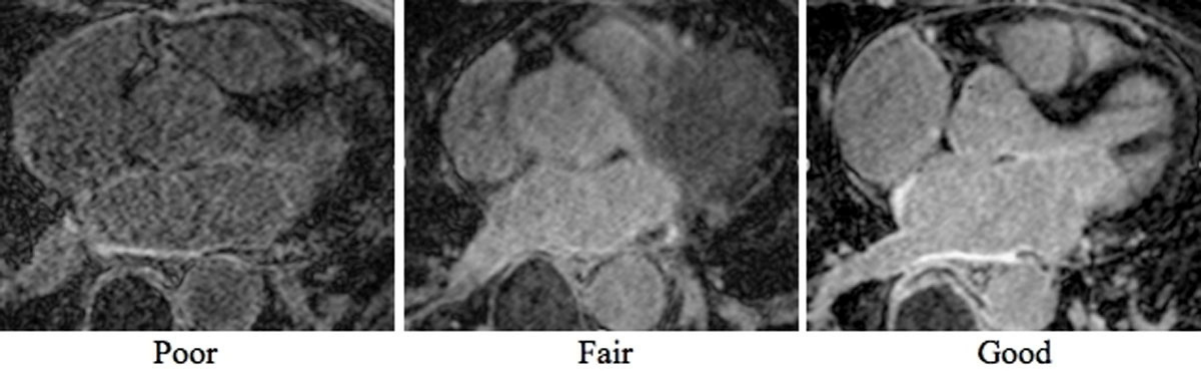
Characteristic images of poor, fair and good quality

Images from 70 patients (48 male) aged 66.2 ± 8.5 years, who underwent radiofrequency ablation for AF and came back for a 3 or 6-month post ablation MRI follow up, were analyzed. All patients were imaged either on a 3T Verio (46) or 1.5T Avanto (24) scanner (Siemens Healthcare, Erlangen, Germany). Dosage of contrast agent (0.1 mmol/kg, Multihance, Bracco Diagnostic Inc., Princeton, NJ), time after injection (15-25 minutes) and spatial resolution of LGE-MRI (1.25x1.25x2.5 mm) were the same for both scanners. The other imaging parameters were optimized for respective field strength (1.5T: TR/TE = 5.1/2.2 ms, flip angle = 20, bandwidth = 290 Hz/pixel; 3T: TR/TE = 3.1/1.4 ms, flip angle = 14, bandwidth = 750 Hz/pixel) to achieve high scar visibility. Data for LGE sequence was sampled during LA diastole and data acquisition window was limited to 15% of cardiac cycle making acquisition time independent on patient heart rate. Two independent reviewers graded the images for clarity and visibility of post ablation scar, on a scale of 1-3 as poor, fair, good respectively (Fig 1). The acquisition times and the respiratory acceptance rate of the images were recorded and correlated with the average image quality (of the 2 independent readers).

## Results

**Figure 2:**
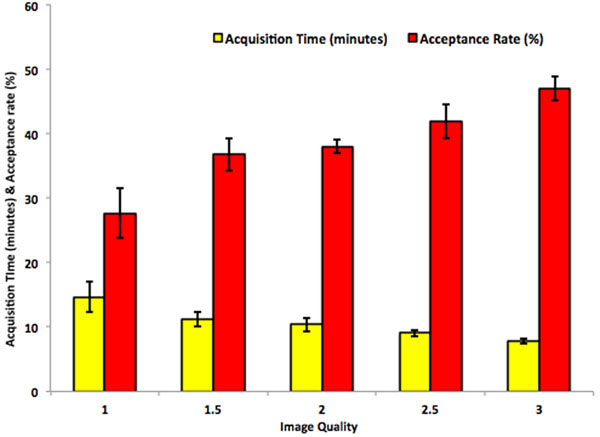
Dependence of image quality on the acquisition time and respiratory navigator acceptance rate

Figure 2 shows the mean and standard deviation for the acquisition time and respiratory navigator acceptance rate for each of the mean values of image quality. It is clearly seen that the image quality is affected by the acquisition time. Acquisition time for good quality images was less than 8 minutes (7.8 ± 1.9) while acquisition time for the poorest images was over 14 minutes (14.7 ± 4.0). The respiratory navigator acceptance rate for good quality images was 47.0 ± 8.9 %, while the poorest images were acquired with an acceptance rate of 27.7 ± 6.6 %. One-way ANOVA performed for the 3 groups of poor, fair, good showed that the differences in acquisition time and acceptance rate are statistically significant (p = 0.0003 and p = 0.0015 respectively).

## Conclusions

This study shows that the quality of LGE-MRI images is highly dependent on the acquisition time and respiratory navigator acceptance rate. The difference in image quality due to the acquisition time and navigator acceptance rate is statistically significant.

## Funding

This work was funded in part by the Ben B and Iris M Margolis foundation.

